# Retraction: Receptor tyrosine kinase AXL is correlated with poor prognosis and induces temozolomide resistance in glioblastoma

**DOI:** 10.1111/cns.13227

**Published:** 2019-10-02

**Authors:** Jia Wang, Jie Zuo, Mao‐De Wang, Wan‐Fu Xie, Xiao‐Bin Bai, Xu‐Dong Ma

**Affiliations:** ^1^ Department of Neurosurgery The First Affiliated Hospital of Xi'an Jiaotong University Xi'an China; ^2^ Center of Brain Science The First Affiliated Hospital of Xi'an Jiaotong University Xi'an China; ^3^ The Second Affiliated Hospital of Xi'an Jiaotong University Xi'an China

## Abstract

Retraction: Receptor tyrosine kinase AXL is correlated with poor prognosis and induces temozolomide resistance in glioblastoma, CNS Neuroscience & Therapeutics 2019, (https://doi.org/10.1111/cns.13227). The above article published online on 02 October 2019 in Wiley Online Library (wileyonlinelibrary.com), has been retracted by agreement between the authors, the journal Editor in Chief Jun Chen, and John Wiley & Sons Ltd. The retraction has been agreed due to unreliable data and consequently its misleading results and conclusions.

ORIGINAL ARTICLE

CNS Neuroscience & Therapeutics Open Access WILEY

RETRACTED

## Receptor tyrosine kinase AXL is correlated with poor prognosis and induces temozolomide resistance in glioblastoma

Jia Wang[1,2] | Jie Zuo[3] | Mao-De Wang[1,2] | Wan-Fu Xie[1] | Xiao-Bin Bai[1] | Xu-Dong Ma[1]

[1]Department of Neurosurgery, The First Affiliated Hospital of Xi'an Jiaotong University, Xi'an, China

[2]Center of Brain Science, The First Affiliated Hospital of Xi'an Jiaotong University, Xi'an, China

[3]The Second Affiliated Hospital of Xi'an Jiaotong University, Xi'an, China

**Funding information**
National Natural Science Foundation of China, Grant/Award Number: 81802502; Project Supported by Natural Science Basic Research Plan in Shaanxi Province of China, Grant/Award Number: 2019JQ-958; Fundamental Research Funds of Xi'an Jiaotong University, Grant/Award Number: 1191329177; Special Foundation for "Class A Subject" of the First Affiliated Hospital of Xi'an Jiaotong University

### Abstract

**Aims:** To investigate the functions and underlying mechanisms of AXL receptor tyrosine kinase (AXL) in tumor proliferation and chemoresistance to temozolomide (TMZ) in glioblastoma (GBM).

**Methods:** With a kinome-wide bioinformatics analysis, AXL was found to be an essential kinase candidate in TMZ chemoresistance promotion. Additionally, the biological functions of AXL in oncogenesis and TMZ resistance were clarified by using qRT-PCR, Western blotting, and in vivo intracranial GBM xenograft models followed the induction of TMZ resistance in U87 or U251 cells. Additionally, immunohistochemistry (IHC) assays were used to investigate the correlation of AXL on the survival of patients with glioma. Finally, the Chou-Talalay model was performed to confirm the synergistic effect of AXL inhibitor TP-0903 with TMZ.

**Results:** Elevated AXL expression significantly correlated with adverse outcomes of patients with glioma, especially patients with GBM. Moreover, AXL knockdown reduced tumorigenesis and TMZ resistance in vitro and in vivo; however, exogenous AXL overexpression induced TMZ resistance in GBM. Lastly, a specific AXL inhibitor, TP-0903, dramatically decreased tumor growth and increased sensitivity to TMZ via a synergistic effect.

**Conclusion:** AXL contributed to chemoresistance to TMZ in GBM and could be used as a novel prognostic biomarker and therapeutic target for GBM.

### 1 | INTRODUCTION

Glioblastoma (GBM) is one of the most frequently diagnosed primary brain malignant tumors in human adults and has a poor prognosis with a 5-year survival rate of <5%, even with aggressive, multimodal therapies.[1] Currently, the comprehensive treatment strategy for GBM consists maximal surgical resection, radiotherapy, and temozolomide (TMZ) chemotherapy.[2] TMZ attenuates GBM growth and thus improves the overall survival of patients with GBM via alkylating/methylating N-7 or O-6 on guanine residues of DNA.[3] However, most patients still suffer tumor recurrence within 7 months.[4] Gradually acquired TMZ resistance of GBM results in tumor progression and recurrence and thus largely limits the benefits of TMZ in patients with GBM.[5] A recent study found that GBM

cells gained resistance to TMZ mainly through O6-methylguanine-DNA-methyltransferase (MGMT)-dependent DNA repair, while the absence of MGMT leads to mismatch repair (MMR) and subsequent DNA double-strand breaks.[6,7] Recent studies have demonstrated that multiple molecules contribute to the acquisition of TMZ resistance.[6,8-10] Therefore, to identify novel potential chemotherapy targets for recurrent GBM, the mechanism of TMZ resistance needs to be further studied.

Accumulating data indicate that kinases are functionally required for multiple biological behaviors in cancer, indicating that kinases could become novel therapeutic targets for cancers.[11] Recent evidence demonstrates that AXL receptor tyrosine kinase (AXL) is essential for a wide range of biological functions, including proliferation, mitosis, migration, invasion, and anti-inflammation in GBM.[12] AXL has been identified as an extracellular kinase with two immunoglobulin-like domains located to fibronectin type III domains.[13] Recent studies have demonstrated that AXL is enriched in pseudopalisading glioma cells around necrotic areas of GBM tumor, while the functional disruption of AXL reduces glioma cell growth and migration in a mouse model.[13,14] Moreover, AXL expression could be related to mesenchymal glioma stem cells, which are considered a more therapy-resistant subtype of GBM and implies a poor prognosis in patients with GBM,[15] providing a powerful evidence for the future therapy development of GBM.

Herein, we analyzed the transcriptome profiling of TMZ-resistant GBM cell lines to identify AXL as a potential druggable kinase in GBM. Furthermore, AXL expression was significantly increased in TMZ-resistant GBM cells, and the exogenous suppression of AXL led to attenuated cell growth and TMZ resistance in GBM. Additionally, the combination of an AXL inhibitor and TMZ dramatically reduced the proliferation and tumorigenesis of TMZ-resistant GBM cells both in vitro and in vivo, which indicated a synergistic effect. In conclusion, AXL was a remarkable biomolecular predictor for TMZ sensitivity and prognosis of patients with GBM and could be a new potential therapeutic target for chemoresistant GBM.

### 2 | METHODS

#### 2.1 | Ethical statement

The animal experiments in this study were approved by the Ethics Committee of the School of Medicine, Xi'an Jiaotong University (No. 2016-085). The acquisition of tumor samples and patient information was approved by the Scientific Ethics Committee of the First Affiliated Hospital of Xi'an Jiaotong University (No. 2016-18). All necessary documents were signed.

#### 2.2 | Reagents

DMEM-F12 medium, fetal bovine serum (FBS), alamarBlue reagent, Alexa Fluor® 488 Annexin V/Dead Cell Apoptosis kit, and PageRuler plus prestained protein ladder were purchased from Thermo Fisher Scientific. Accutase buffer, RIPA lysis buffer, and inhibitors for phosphatase were purchased from Merck KGaA. Bradford reagent and Reverse Transcription SuperMix were purchased from Bio-Rad. TP-0903 was purchased from AbMole BioScience (Catalog no. M8915). TMZ was provided by Tasly Group Co, Ltd.

#### 2.3 | Antibodies

Anti-AXL primary antibodies were purchased from Cell Signaling Technology (catalog No. #8661, for western blotting) and from Invitrogen (catalog No. PA5-28850, for immunohistochemistry). Anti-phospho-AXL primary antibody was purchased from R&D Systems (catalog No. AF2228). Anti-rabbit IgG was purchased from Abcam (catalog No. ab171870) and used as a negative control. Horseradish peroxidase-conjugated goat anti-rabbit IgG (catalog No. ab97051) and goat anti-mouse IgG (catalog No. ab205719) were purchased from Abcam and used as secondary antibodies.

#### 2.4 | Bioinformatics analysis

Gene expression data were extracted from previously published databases that compared TMZ-resistant glioma stem cells with the original cells (Tso et al,[16] GSE 68029, 2015). Hierarchical bi-clustering analysis was used to investigate the targeting gene expression with Cluster 3.0. Next, euclidean distance and average linkage were used as similarity metric and clustering methods, respectively. Gene expression between the naïve and resistant GBM cells was presented as fold changes.

#### 2.5 | In vitro cell culture

U87 cell line was purchased from BeNa Culture Collection. U251, T98MG, SHG-44, A172, and normal human astrocytes (NHAs) were obtained from the First Affiliated Hospital of Xi'an Jiaotong University. Cells were cultured in DMEM-F12 medium containing 10% FBS under 37°C with 5% $CO_2$. Fresh medium was added every 2-3 days. The number of cells was measured using a cell counter with trypan blue, and the cells were seeded at a density of $10^6$ cells/10 mL.

#### 2.6 | Induction of TMZ resistance in GBM cells

U87 or U251 cells were cultured with DMEM-F12 containing 10% FBS at 37°C with 5% $CO_2$. Cells were treated with TMZ at a starting dose of 100 μM. Medium containing TMZ (100 μM) was replaced every 24 hours for the first 5 days. Afterward, TMZ dose was increased every 2 weeks. After 3 months of culture, the maintenance dose of TMZ was increased to 500 μM.

#### 2.7 | In vitro cell proliferation assay

Single cell suspensions were seeded into 96-well plates with a density of 1000 cells per well with 100 μL of fresh medium. Cell number was calculated using alamarBlue at days 0, 2, 4, 6, and 8 according to the manufacturer's protocol.

RETRACTED

RETRACTED

#### 2.8 | In vitro cell viability assay

Single cell suspensions were seeded into 96-well plates (2000 cells/100 μL each well) and cultured for 12 hours at 37°C with 5% $CO_2$; then, 100 μL of fresh medium containing TMZ or TP-0903 at different concentrations was added for 3 days. The cell number was measured using alamarBlue as previously described. The IC50 was calculated using SPSS 19.0 software. The synergism or antagonism of TMZ and TP-0903 was tested using COMPUSYN 2.0 with the Chou-Talalay model.[17]

#### 2.9 | Quantitative RT-PCR (qRT-PCR)

qRT-PCR analysis was performed as previously described.[18] The sequences of the primers used in this study are as follows: AXL-forward: GTTTGGAGCTGTGATGGAAGGC; AXL-Reverse: CGCTTCA CTCAGGAAATCCTCC;GAPDH-forward:GAAGGTGAAGGTCGGAG TCA; GAPDH-reverse: TTGAGGTCAATGAAGGGGTC.

The primers were confirmed to be 100% amplified before analysis, and GAPAD was used as an internal control to normalize the expression. The relative quantification of cDNAs to GAPDH was determined via the $2^{-\Delta\Delta Ct}$method.

#### 2.10 | Western blotting

Cells were collected and prepared with RIPA buffer according to the manufacturer's instruction. Protein concentration was measured using the Bradford method. Western blotting analysis was performed as previously described.[18] β-actin served as a loading control. The Western blotting results were quantified using ImageJ and are presented as histograms.

#### 2.11 | Immunohistochemistry (IHC)

Patient samples used in this study were pathologically diagnosed after surgery. IHC was performed as previously described.[18] The German IHC scoring (GIS) method was used to evaluate AXL expression as previously described.[18] Immunoreactivity score = positive cell score × staining intensity score. The positive cell score was calculated as follows: 0, negative staining; 1, positive cell ratio <10%; 2, positive cell ratio 11%-50%; 3, positive cell ratio 51%-80%; and 4, positive cell ratio >80%. Staining intensity score was graded as follows: 0, negative staining; 1, weakly positive; 2, moderately positive; and 3, strongly positive. An immunoreactivity score more than 3 was considered high expression.

#### 2.12 | Lentivirus production and transduction

Lentivirus production and transduction were performed as described previously.[18] The sequence of shAXL cloned into the lentiviral plasmid is as follows: shAXL#1, GCGGTCTGCATGAAGGAATTT; and shAXL#2, GCTGTGAAGACGATGAAGATT. The pLenti-GIII-CMV-AXL lentivirus was purchased from Applied Biological Materials (Cat log no. LVP086248, Richmond, BC, Canada).

GBM cells were dissociated mechanically with Accutase before lentivirus infection and were seeded into 96-well plates at a density of 10 000 cells/100 mL per well. A total of 20 μL of lentivirus were added to the medium, and the medium was renewed after 12 hours. The efficiency of knockdown or overexpression was confirmed by qRT-PCR and Western blotting.

#### 2.13 | Flow cytometry

Flow cytometry analysis was performed as previously described.[18] Pretransfected GBM cells were exposed to TMZ with or without TP0903 for 3 days. Cell apoptosis was evaluated by using Alexa Fluor® 488 Annexin V/Dead Cell Apoptosis kit following the manufacturer's instruction.

#### 2.14 | In vivo intracranial xenograft tumor model

Female nude mice (6 weeks old) were prepared for the in vivo transplantation of GBM cells. The prepared GBM cell suspension (infected with shNT, shAXL, or AXL overexpression lentivirus) was diluted to $10^5$ cells in 2 μL of PBS and then injected into the mouse brains as previously described.[18,19] The mice were monitored daily until the following symptoms could be observed: arched back, leg paralysis, unsteady gait, or bodyweight loss of more than 10%. TMZ (50 mg/kg/d) and TP-0903 (20 mg/kg/d) were administered by tail vein injection at 7 days after the injection of glioma cells.

#### 2.15 | Statistical analysis

All results in this study are presented as the mean ± SD. Statistical significance between two groups was compared with 2-tailed *t* tests. Statistical significance for multiple groups was compared with one-way analysis of variance (one-way ANOVA) followed with Dunnett's posttest. Kaplan-Meier plots were compared with log-rank analysis. All statistical analyses were performed using GraphPad Prism 6.0 or SPSS 19.0 software. $P < .05$ was considered significant.

### 3 | RESULTS

#### 3.1 | AXL expression was significantly elevated in GBM

To explore the key regulator and molecular mechanism of acquired TMZ resistance in glioma, hierarchical bi-clustering was performed by using a previously published GEO database (GSE 68029, 2015).[16] Among the 668 known kinase-encoding genes, AXL was one of the most top-ranked kinase-encoding genes in TMZ-resistant GBM cells compared with their naïve control cells (Figure 1A), demonstrating that AXL could be essential for glioma to gain TMZ resistance. Additionally, AXL was significantly enriched in GBM, which is considered the most lethal type of glioma (Figure 1B). GBM cells can be divided into several subtypes based on the epigenetic signatures that are associated with completely different biological behaviors, including tumor

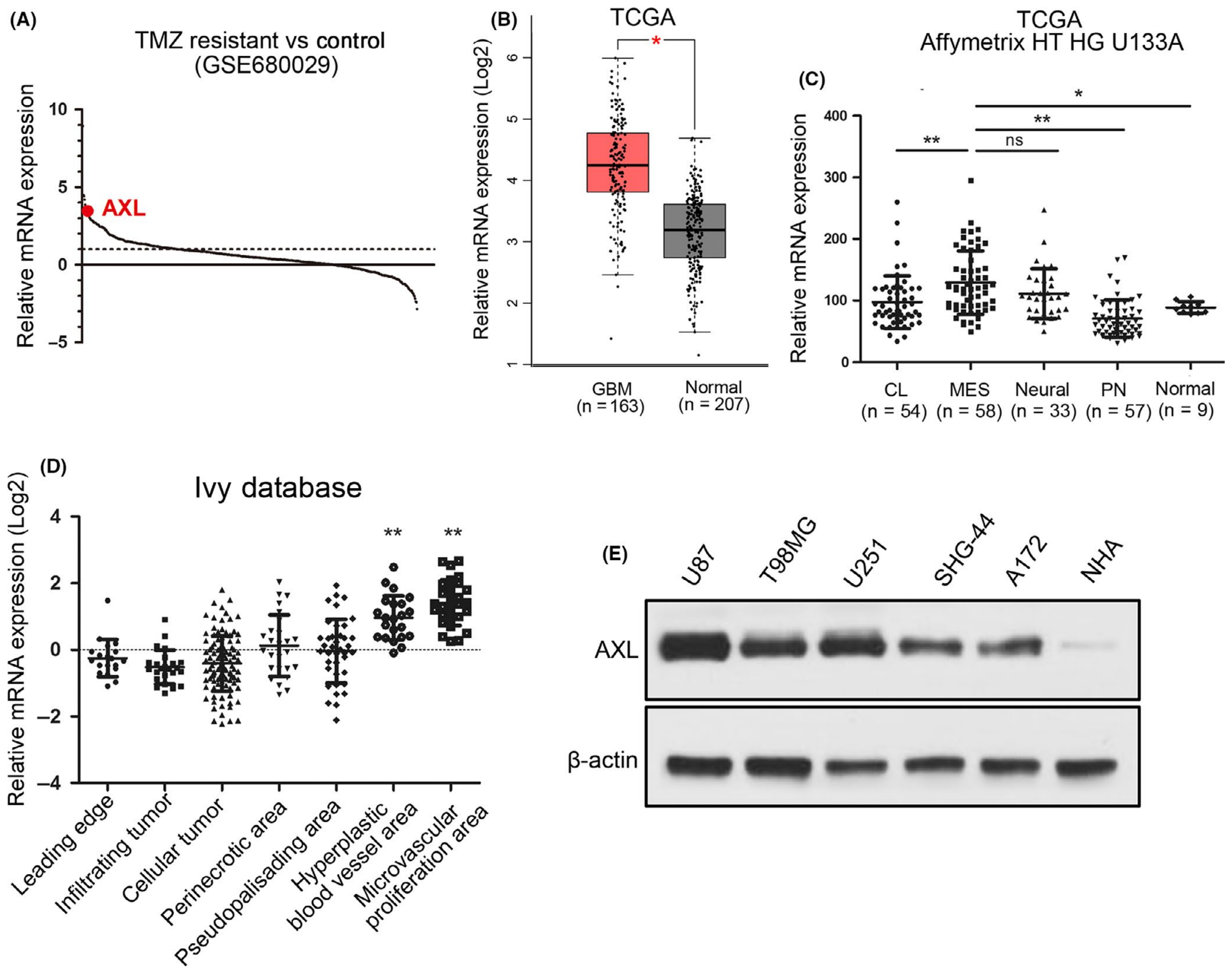

**FIGURE 1** AXL was highly expressed in GBM. A, Genome-wide transcriptome analysis (GSE 68029) indicated that AXL was one of the top-ranked kinase-encoding genes in TMZ-resistant GBM compared with naïve cells. B, Gene expression analysis using TCGA database showed that AXL was elevated in GBM compared with nontumor tissue (*$P < .05$, *t* test). C, Gene expression analysis with TCGA database indicated that AXL was highly expressed in mesenchymal GBM samples (*$P < .05$, **$P < .01$, ns $P > .05$, one-way ANOVA followed by Dunnett's posttest). D, Relative mRNA expression for AXL was increased in the hyperplastic blood vessels and microvascular proliferation area Ivy GBM dataset (*$P < .05$, **$P < .01$, one-way ANOVA followed by Dunnett's posttest). E, Western blotting analysis of AXL expression in GBM cell lines compared with NHA cells. β-actin served as a control proliferation, recurrence, and therapy resistance. Therefore, we compared AXL expression among the different GBM subtypes. The results indicated that AXL expression was markedly enriched in the mesenchymal subtype, which is considered to be more therapy resistant and leads to tumor recurrence (Figure 1C). Furthermore, when analyzing the Ivy dataset (http://glioblastoma.alleninstitute.org/static/home), an increased AXL expression could be observed in hyperplastic blood vessel area and the microvascular proliferation area, which was shown to be related to tumor recurrence in GBM (Figure 1D). Lastly, Western blotting was performed using 5 GBM lines (U87, U251, T98MG, SHG-44, and A172), while NHA was used as a nontumor control. The results showed significantly increased AXL expression in GBM cell compared with NHA cells (Figures 1E and S1A). All together, these results showed that AXL was elevated in GBM and might be essential for therapy resistance and tumor recurrence.

#### 3.2 | Elevated AXL expression was correlated with poor prognosis in patients with GBM

As mentioned previously, these results suggested that AXL may be a biomarker for GBM. To evaluate this possibility, an analysis of overall survival was performed among all 514 available glioma patients from TCGA database. The results indicated that the postsurgical survival of patients with lower AXL expression was significantly prolonged compared with those with higher AXL expression (Figure 2A). We next assessed AXL expression in GBM by analyzing the Rembrandt database. The results demonstrated that AXL was markedly increased in GBM compared with low-grade gliomas (Figure 2B). Additionally, IHC was performed to examine AXL expression in 68 glioma tumor tissues collected from patients received surgical resection from 2008 to 2017 in the Department of Neurosurgery, the First Affiliated Hospital of Xi'an Jiaotong University.

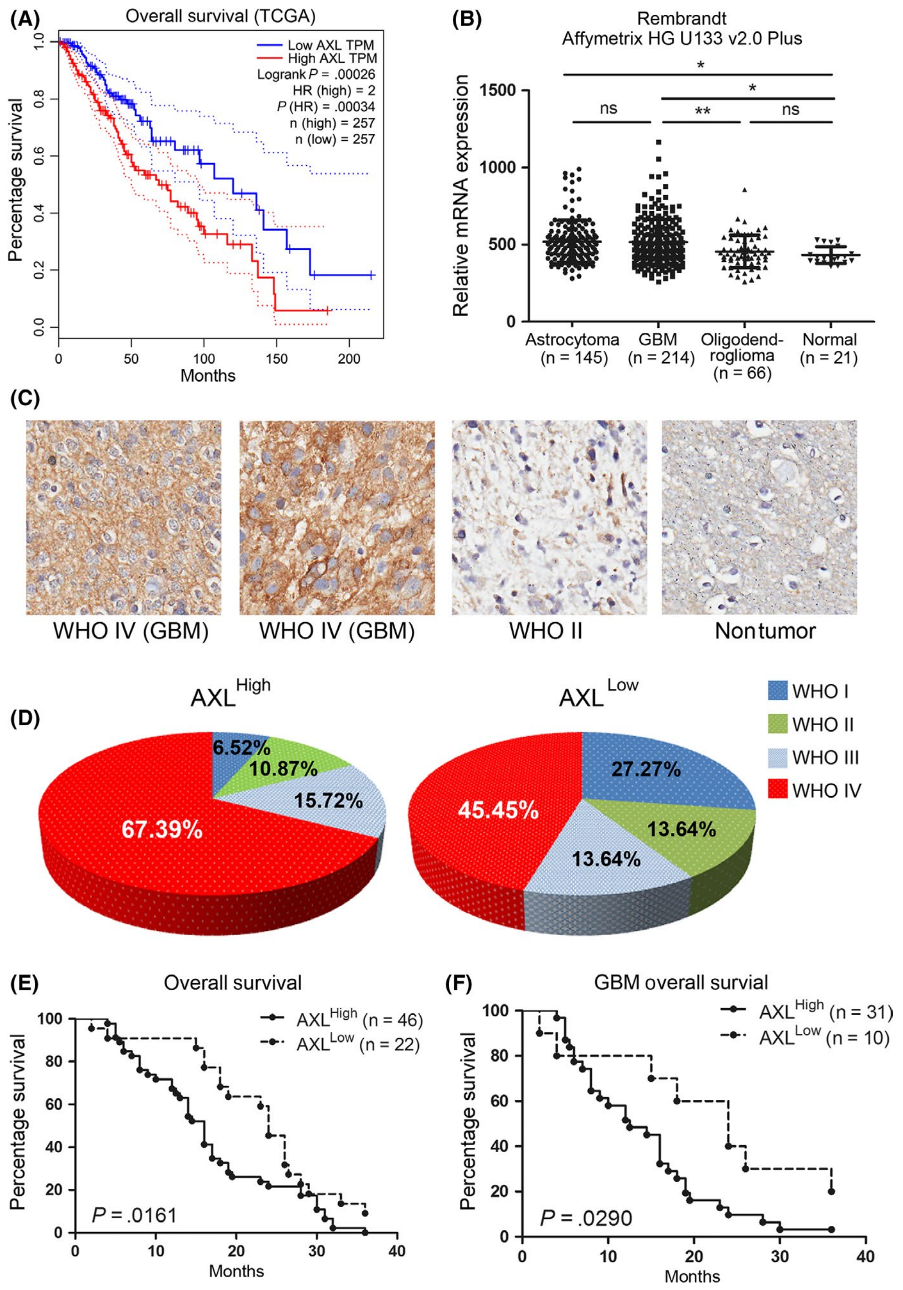

**FIGURE 2** AXL was a clinically relevant molecular target of GBM. A, Kaplan-Meier survival analysis with TCGA database indicated the inverted correlation between AXL mRNA and overall survival of patients with GBM ($P < .01$, n = 257 for the AXL-upregulated group, n = 257 for AXL-downregulated group, with log-rank test). B, Gene expression analysis with Rembrandt database showed that AXL was elevated in GBM samples compared with nontumor tissues (*$P < .05$, **$P < .01$, ns $P > .05$, one-way ANOVA followed by Dunnett's posttest). C, Representative immunohistochemical images of AXL in glioma samples. Nontumor tissues from epilepsy surgery were used as a negative control. D, AXL was highly enriched in high grade glioma samples (WHO III-IV) compared with low-grade glioma samples (WHO I-II). E, Kaplan-Meier analysis indicated a prolonged overall survival in glioma samples with lower AXL expression compared with those with higher AXL expression among all 68 patients with glioma ($P = .0161$, with log-rank test). F, Kaplan-Meier analysis indicated a prolonged overall survival in glioma samples with lower AXL expression compared with those with higher AXL expression among all 41 patients with GBM ($P = .029$, with log-rank test)

AXL was found to be expressed in the cytoplasm as well as the cell membrane of tumor cells (Figure 2C). The GIS score showed that AXL was markedly enriched in GBM compared with low-grade glioma samples (Figure 2D). With regard to patient survival, the patients with gliomas with low AXL expression presented a longer overall survival compared with those whose gliomas expressed high levels of AXL (Figure 2E). Similar results were achieved when we specifically focused on patients with GBM (Figure 2F). Collectively, the results showed the possibility that AXL may be a specific clinically relevant kinase for GBM.

### 3.3 | AXL was increased in TMZ-resistant GBMs

To thoroughly study the functional role of AXL in acquired resistance to TMZ in GBM, we established TMZ-resistant in vitro *cultures with* U87 and U251 according to the previous publications.[20,21] After culturing with TMZ-containing medium for 3 months, U87 and U251 cells gained stable TMZ resistance compared with their naïve controls (Figure 3A,B). qRT-PCR analysis was performed using the TMZ-resistant GBM cell lines and the results indicated dramatically increased AXL expression in TMZ-resistant population of U87 and U251 cells (Figure 3C). Moreover, Western blotting results also showed upregulated AXL and phospho-AXL in the TMZ-resistant population of U87 as well as U251 cells (Figures 3D, S1B and S1C).

### 3.4 | AXL contributed to acquired TMZ resistance in GBM

For further assessment of the functional role of AXL in GBM, the GBM cell lines U87 and U251 were transduced with 2 lentiviral shRNA clones for AXL (shAXL #1 and shAXL #2) or nontargeting

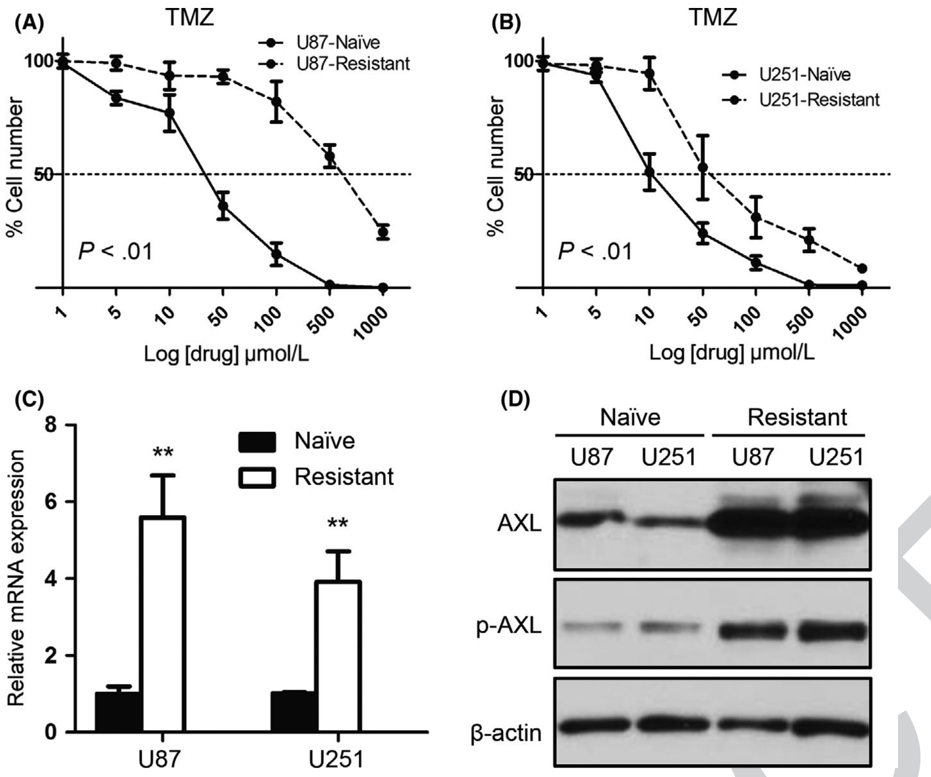

**FIGURE 3** Increased AXL could be observed in TMZ-resistant GBMs. A, In vitro toxicity assay for TMZ in U87 naïve cells and U87 TMZ-resistant cells (IC50: 40.15 μM for U87 naïve and 611.3 μM for U87-resistant cells, n = 6, $P < .01$, with one-way ANOVA, cells were treated with TMZ for 3 d). B, In vitro toxicity assay for TMZ in U251 naïve cells and U251 TMZ-resistant cells (IC50: 9.802 μM for U87 naïve and 39.94 μM for U87 TMZ-resistant cells, n = 6, $P < .01$, one-way ANOVA, cells were treated with TMZ for 3 d). C, qRT-PCR results indicated that AXL expression was substantially increased in TMZ-resistant U87 or U251 cells (**$P < .01$, *t* test). D, Western blotting analysis showed that AXL protein was increased in TMZ-resistant U87 or U251 cells. β-actin served as a control control lentivirus (shNT). Both qRT-PCR and Western blotting analysis showed dramatic downregulation of AXL at the mRNA level (Figures 4A, S1B and S1D). Additionally, to evaluate the function of AXL on TMZ resistance, U87 TMZ-resistant cells were transduced with shNT or shAXL lentivirus followed by TMZ treatment at 300 μM. An in vitro cell growth assay exhibited decreased cell proliferation and enhanced TMZ sensitivity in U87 TMZ-resistant cells after transduction with shAXL lentivirus (Figure 4C). Similarly, flow cytometry assays for apoptosis were performed with shAXL or shNT pretransduced U87 TMZ-resistant cells treated with or without TMZ (300 μM). The population of cells undergoing either early apoptosis ($AV^+PI^-$) or late apoptosis ($AV^+PI^+$) was markedly increased when cells received TMZ treatment combined with AXL silencing compared with those that received TMZ alone (Figure 4D). Next, the function of ALX knockdown on in vivo tumorigenesis was investigated using intracranial xenografted mice models. The results indicated that the mice with xenografts of shNT-transduced U87 TMZ-resistant cells rapidly presented with tumor-related symptoms compared with those transplanted with shAXL-transduced U87 TMZ-resistant cells combined with TMZ treatment (Figure 4E), showing a potent anti-TMZ resistance effects of AXL silencing in GBM cells.

To clarify the molecular mechanism of AXL-dependent TMZ resistance, an artificial overexpression of AXL via lentiviral infection was performed in U87 GBM cells. qRT-PCR and Western blotting analysis confirmed that both AXL and phosphorylated AXL were markedly increased in AXL-overexpressed U87 cells (Figures 4F,G and S1E). An in vitro cell growth assay indicated that TMZ resistance was obviously increased after exogenous AXL overexpression (Figure 4H). Moreover, U87 cells with or without ALX overexpression were treated with TMZ, and apoptosis was analyzed. The results showed enhanced TMZ resistance after AXL overexpression (Figure 4I), demonstrating that AXL contributed to acquired TMZ resistance in GBM.

#### 3.5 | AXL inhibitor attenuated TMZ resistance of GBM cells via synergistic effects

AXL was identified in this study as an essential regulator of acquired TMZ resistance in GBM. Thus, to characterize the efficacy of AXL inhibitor in GBM treatment, TP-0903 was used as a specific AXL inhibitor in this study. Firstly, we found that U87 TMZ-resistant cells showed the same response to TP-0903, indicating that AXL inhibitor was still efficient for TMZ-resistant GBM (Figure 5A). Moreover, an in vitro proliferation assay indicated that TP-0903 attenuated the growth of TMZ-resistant U87 cells, and more importantly, the gained TMZ resistance was significantly reversed by TP-0903 (Figure 5B). Flow cytometry showed that TP-0903 treatment increased the proportion of apoptosis and enhanced the cytotoxicity of TMZ on U87 TMZ-resistant cells (Figure 5C). Next, we investigated the effects of TP-0903 on in vivo tumorigenesis. Intracranial xenografted mouse models were used, and the treatment of TMZ and TP-0903 was initiated at 7 days after implantation with U87 TMZ-resistant cells and continued for 10 days by tail vein injection. As expected, very limited tumor growth suppression was noted when mice were treated with TMZ alone; however, dramatically reduced tumor growth and prolonged survival could be observed when TMZ was combined with TP-0903. These results demonstrate that the combination of the AXL inhibitor and TMZ attenuated tumor proliferation and the tumorigenesis of the TMZ-resistant GBM cells (Figure 5D).

RETRACTED

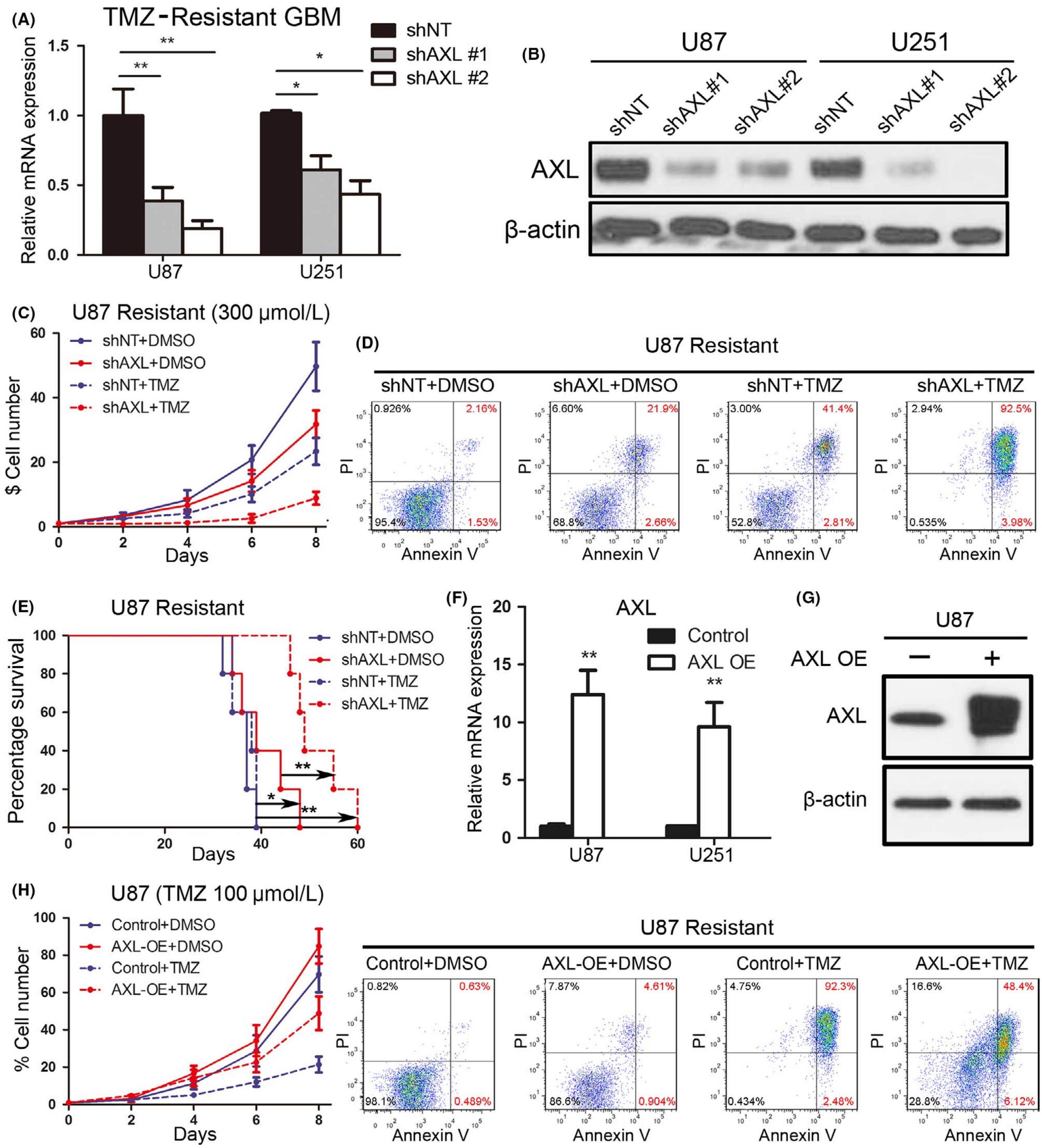

**FIGURE 4** AXL contributed to acquired TMZ resistance in GBM. A, qRT-PCR analysis for AXL expression in U87 or U251 TMZ-resistant cells transduced with shRNAs against AXL (shAXL #1 and shAXL #2) or nontargeting lentivirus (shNT) (*$P < .05$, **$P < .01$, with one-way ANOVA followed by Dunnett's posttest). B, Western blotting for AXL in U87 or U251 TMZ-resistant cells transduced with shRNAs against AXL (shAXL #1 and shAXL #2) or nontargeting lentivirus (shNT). β-actin served as a control. C, In vitro cell proliferation assay for AXL knockdown combined with TMZ treatment in U87 TMZ-resistant cells (*$P < .05$, one-way ANOVA). D, Flow cytometry analysis for apoptosis with Annexin V antibody and propidium iodide using U87 TMZ-resistant cells pre-infected with shAXL or shNT lentivirus followed by the presence of absence of TMZ treatment (300 μM, treated for 3 d). E, Kaplan-Meier analysis for mice after intracranial xenografts of U87 TMZ-resistant cells pretreated with shAXL or shNT lentivirus followed by continuous 10-d TMZ (50 mg/kg/d) treatment or placebo (DMSO) by tail vein injection (*$P < .05$, **$P < .01$, log-rank test). F, qRT-PCR analysis of AXL in U87 or U251 cells transduced with AXL overexpression lentivirus (AXL OE) or control lentivirus (Control) (**$P < .01$, *t* test). G, Western blotting analysis of AXL and phosphorylated AXL in U87 or U251 cells transduced with AXL overexpression lentivirus (AXL OE) or control lentivirus (Control). β-actin served as a control. H, In vitro cell proliferation assay for AXL-overexpressed U87 cells treated with TMZ (*$P < .05$, one-way ANOVA). I, Flow cytometry analysis for apoptosis for AXL-overexpressed U87 cells treated with TMZ using Annexin V and propidium iodide (100 μM, treated for 3 d)

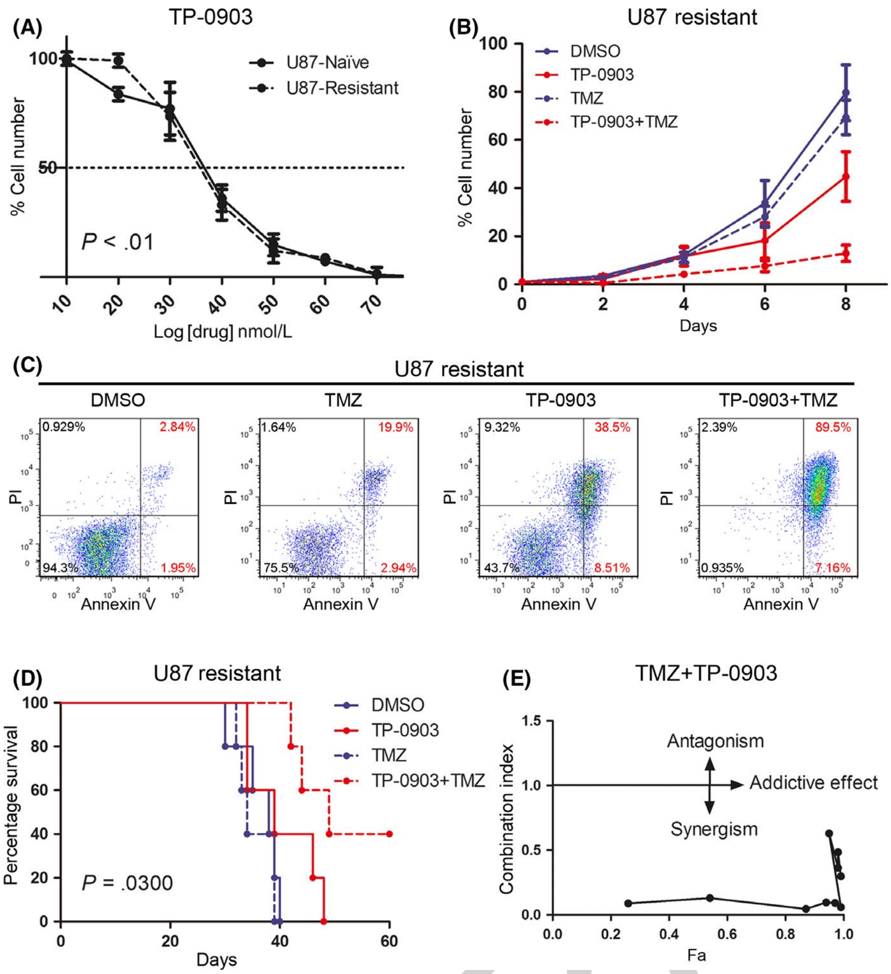

**FIGURE 5** AXL inhibitor reduced TMZ resistance of GBM cells via a synergistic effect. A, In vitro toxicity assay for TP-0903 in U87 naïve cells and U87 TMZ-resistant cells (IC50: 37.06 nM for U87 naïve and 35.15 μM for U87 TMZ-resistant cells, n = 6, *P* < .01, one-way ANOVA, treated with TP-0903 for 3 d). B, In vitro cell proliferation assay for TP-0903 combined with TMZ treatment in U87 TMZ-resistant cells (n = 6, **P* < .05, one-way ANOVA, treated with TP-0903 at 40 nM for 3 d). C, Flow cytometry analysis for TP-0903 combined with TMZ treatment in U87 TMZ-resistant cells (TMZ 300 μM, TP-0903 40 nM, treated for 3 d). D, Kaplan-Meier analysis for mice after implantation of intracranial xenografts with U87 TMZ-resistant cells followed with continuously by 10 d of TMZ treatment (TMZ 50 mg/kg/d, TP-0903 20 mg/kg/d) or placebo (DMSO) via tail vein injection (*P* = .03, with log-rank test). E, Chou-Talalay model showed the commination index (CI) of TP-0903 and TMZ, which indicated that TP-0903 had synergistic effects with TMZ on U87 TMZ-resistant GBM cells

| TP-0903 (nmol/L) | Cell Growth Inhibition (%) | TMZ (μg/mL) 0.01 | 0.05 | 0.1 | 0.5 | 1 | 2 | 4 | 6 | 8 | 10 |
|---|---|---|---|---|---|---|---|---|---|---|---|
| | | 1.23 | 3.12 | 5.44 | 13.43 | 21.34 | 40.97 | 54.81 | 69.17 | 88.65 | 95.89 |
| 0.1 | 3.21 | 26.39 | | | | | | | | | |
| 0.5 | 12.14 | | 54.54 | | | | | | | | |
| 1 | 23.32 | | | 87.34 | | | | | | | |
| 5 | 51.17 | | | | 94.67 | | | | | | |
| 10 | 67.65 | | | | | 97.88 | | | | | |
| 20 | 79.45 | | | | | | 99.87 | | | | |
| 40 | 89.14 | | | | | | | 95.43 | | | |
| 60 | 91.08 | | | | | | | | 98.12 | | |
| 80 | 93.44 | | | | | | | | | 98.87 | |
| 100 | 99.59 | | | | | | | | | | 99.29 |

**FIGURE 6** Chou-Talalay analysis for TMZ and TP-0903 in U87 TMZ-resistant GBM cells

To further clarify whether AXL inhibition reduced tumor growth and resistance to TMZ through additive or synergistic effects with TMZ, Chou-Talalay methods were applied as previously reported.[17] U87 TMZ-resistant cells were treated with TMZ, TP-0903, or a combination of these 2 drugs at different doses, and the inhibition of cell growth was measured at day 3. The data showed that the combination index (CI) of TMZ and TP-0903 was <1, which indicates that AXL inhibition has synergistic effects with TMZ treatment on TMZ-resistant U87 cells (Figure 5E). Detailed information regarding the Chou-Talalay test is shown in Figure 6.

### 4 | DISCUSSION

Accumulating evidence demonstrated that kinase-dependent activation of acquired resistance to radiotherapy and chemotherapy is essential for the recurrence and lethal mortality of GBM.[22,23] Among the known kinase-encoding genes, AXL has been proved to be responsible for various biological behaviors, such as cell proliferation, invasion, and tumor proliferation, in a wide range of human malignant cancers, such as breast cancer, pancreatic cancer, ovarian cancer, and lung cancer, etc[24-27] AXL belongs to the TAM (TYRO3-, AXL-, and MER-TK) subfamily of receptor tyrosine kinases that share structural homology within the kinase domain.[28] A previous study has demonstrated that aberrantly elevated AXL expression could be observed in GBM; moreover, the suppression of AXL reduces GBM cell proliferation and tumor migration.[15] Interestingly, AXL knockdown has shown more efficient inhibition in mesenchymal GBM specifically, which has been shown to be a more malignant and radio-resistant subtype.[15] In addition, the clinical relevance of AXL has been investigated in a wide range of previous studies.[15,29,30] Similarly, our study indicated that increased AXL expression contributed to the poor prognosis of glioma, especially GBM, which has been shown to be more resistant to existing therapies. Moreover, our results indicated that AXL expression was elevated in TMZ-resistant GBM cells compared with naïve GBM cells and closely correlated with TMZ resistance in GBM. Another novel finding of our study was that suppression of AXL through either lentiviral transfection or inhibitor resulted in decreased tumorigenesis and reduced TMZ resistance of GBM cells in vitro and in vivo, indicating that AXL could be a clinical therapeutic target for GBM. Moreover, the TMZ inhibitor TP-0903 significantly reduced tumor growth via a synergistic effect with TMZ. Our findings indicated that AXL promoted tumorigenesis and chemoresistance to TMZ in GBM and could be used as a prognostic biomarker for patients with GBM; additionally, AXL inhibitors could be developed to sensitize GBM cells to TMZ and improve survival for patients with GBM. To this end, a new AXL inhibitor with higher efficacy and fewer side effects should be designed and generated, and clinical trials, especially multicenter clinical randomized controlled trials, should be performed in the future.

A previous study has indicated that AXL functions as an oncogene mainly through the phosphorylation of Tyr779 and Tyr691 within the functional domain of AXL kinase.[14] Phosphorylated AXL is enriched in characteristic area of GBM tumors, such as hypercellular zones, pseudo-palisades area, and vascular proliferates.[31] Similarly, by analyzing the Ivy glioma database, an increased AXL expression could be observed in the hyperplastic blood vessels and microvascular proliferation area compared with other areas of the tumor. As GBM tumorigenesis and invasion is highly dependent on these specific areas,[32] these results raise up the possibility that AXL activation based on phosphorylation is essential for acquired chemoresistance to TMZ in GBM. However, the molecular mechanism still needs to be further investigated. In addition, the activation mechanisms of AXL and the underlying molecular signaling was not clearly clarified in this study; however, further research for investigating the transcriptional regulation and phosphorylation mechanism of AXL in TMZ resistance of GBM should be designed and performed in the future.

In conclusion, we showed through a kinome-wide analysis that AXL was one of the most elevated kinase-encoding genes in TMZ-resistant GBM cells. Additionally, elevated AXL expression indicated a poor prognosis of patients with GBM. Moreover, AXL knockdown reduced tumorigenesis and chemoresistance in U87 TMZ-resistant cells in vitro and in vivo; however, exogenous AXL overexpression induced TMZ resistance in GBM. Lastly, a specific AXL inhibitor TP-0903 dramatically decreased tumor growth and increased sensitivity to TMZ via synergistic effects. AXL contributed to acquire TMZ resistance in GBM and could be used as a novel prognostic factor and therapeutic target for GBM.

### 5 | CONCLUSION

AXL contributed to tumorigenesis as well as the acquired TMZ resistance in GBM and could become a novel prognostic biomarker and therapeutic target for GBM.

#### ACKNOWLEDGMENTS

The authors thank all members of the Department of Neurosurgery and the Center of Brain Science of the First Affiliated Hospital of Xi'an Jiaotong University. This study was financially supported by the National Natural Science Foundation of China (no. 81802502), the Project Supported by Natural Science Basic Research Plan in Shaanxi Province of China (no. 2019JQ-958), the Fundamental Research Funds of Xi'an Jiaotong University (no. 1191329177), and the Special Foundation for "Class A Subject" of the First Affiliated Hospital of Xi'an Jiaotong University.

#### CONFLICT OF INTEREST

The authors declared that they have no conflicts of interest to this work.

#### ORCID

*Jia Wang* https://orcid.org/0000-0002-4746-035X

RETRACTED

### SUPPORTING INFORMATION

Additional supporting information may be found online in the Supporting Information section at the end of the article.

**How to cite this article:** Wang J, Zuo J, Wang M-D, Xie W-F, Bai X-B, Ma X-D. Receptor tyrosine kinase AXL is correlated with poor prognosis and induces temozolomide resistance in glioblastoma. *CNS Neurosci Ther*. 2020;26:1–10. https://doi.org/10.1111/cns.13227

RETRACTED
